# Moving from intervention management to disease management: a qualitative study exploring a systems approach to health technology assessment in Canada

**DOI:** 10.1017/S0266462323002696

**Published:** 2023-11-06

**Authors:** Marina Richardson, Beate Sander, Nick Daneman, Chloe Mighton, Fiona A. Miller

**Affiliations:** 1 Institute of Health Policy, Management and Evaluation, University of Toronto, Toronto, ON, Canada; 2 Toronto Health Economics and Technology Assessment (THETA) Collaborative, University Health Network, Toronto, ON, Canada; 3 ICES, Toronto, ON, Canada; 4 Public Health Ontario, Toronto, ON, Canada; 5 Division of Infectious Diseases, Sunnybrook Health Sciences Centre, Toronto, ON, Canada

**Keywords:** qualitative research, health technology, health technology assessment, disease management, health policy

## Abstract

**Objectives:**

Health technology assessment (HTA) traditionally informs decision making for single health technologies, which could lead to ill-informed decisions, suboptimal care, and system inefficiencies. We explored opportunities for conceptualizing the decision space in HTA as a disease management question versus an intervention management question.

**Methods:**

Semistructured interviews were conducted between April 2022 and October 2022 with purposefully selected individuals from national and provincial HTA agencies and related organizations in Canada. We conducted manual line by line coding of data informed by our interview guide and sensitizing concepts from the literature. One author coded the data, and findings were independently verified by a second author who coded a subset of transcripts

**Results:**

Twenty-four invitations were distributed, and eighteen individuals agreed to participate. A disease management approach to HTA was differentiated from traditional approaches as being disease-based, multi-interventional, and dynamic. There was general support for an explicit care pathway approach to HTA by informing discussions around patient choice and suboptimal care, creating a space where decision makers can collaborate on shared objectives, and in setting up a platform for open dialogue about managing high-cost and high-severity diseases. There are opportunities for a care pathway approach to be implemented that build on the strengths of the existing HTA system in Canada.

**Conclusions:**

A disease management approach may enhance the impact of HTA by supporting dynamic decision making that could better inform a proactive, resilient, and sustainable healthcare system in Canada.

## Introduction

Health technology assessment (HTA) is an evidence-based, multidisciplinary process that informs decision making by providing insight into the value of a health technology (1). HTA has an established role in the management of health technologies which aims to support a high-quality, efficient, and equitable health system (1). Traditionally, HTA determines if a single health technology (i.e., a medicine, device, test, procedure, or program) offers good value to the healthcare system and at what price.

HTAs respond to the needs of healthcare system decision makers, generally focused on individual coverage or reimbursement portfolios (e.g., outpatient drug coverage decisions are independent of public health intervention decision making). In Canada, CADTH pharmaceutical review committees issue recommendations to provincial and territorial drug programs and separately, and the Public Health Agency of Canada’s (PHAC) National Advisory Committee on Immunization (NACI) issues recommendations to provincial and territorial public health programs. Increasingly, there is recognition that decisions made in isolation from cognate considerations (e.g., isolating decisions about drug treatments for condition A from decisions about preventative treatments for condition A) could lead to ill-informed decisions, suboptimal care, and system inefficiencies (2). Further, decision makers are increasingly asking system-relevant questions that move beyond the traditional mold of assessing a single health technology and instead considering treatment sequencing, combination therapies, companion diagnostics (3), or how best to respond to public health emergencies (4). Innovation in the application of HTA processes and methods are needed to respond to the shift in policy making challenges.

In response to the needs of individual decision makers, the decision space in HTA is classically concerned with intervention management, and often more narrowly, technology adoption. An alternate way to approach the HTA decision, and one that may respond to shifting priorities, is one of disease management (5). Considering the disease as the unit of analysis for an HTA review may offer opportunities to consider a broader range of alternative interventions (i.e., primary prevention, screening, diagnostics, treatment, management), allow for systematic consideration of upstream interventions, and allow the system to question the optimal management of a disease versus a piecemeal or reactive approach that is characteristic of the current environment (6).

Our objective was to explore opportunities to implement a disease management approach to health technology assessment (HTA) in Canada. Specifically, we sought to understand how HTA experts perceive the value of using a disease management approach to HTA in Canada and the anticipated barriers and facilitators to using this approach.

## Methods

Participants were purposefully sampled (7) from national and provincial HTA agencies and related organizations in Canada. We sought to speak with individuals with sufficient knowledge of the HTA system in Canada to be able to critically assess the implications of a shift in HTA from one of intervention management to that of disease management. We anticipated that perspectives would vary depending on organizational affiliation (i.e., federal or provincial, and HTA organization, HTA committee, or health system decision maker), primary field of expertise (i.e., ethics, economics, clinical), intervention domain (i.e., prevention, screening, diagnostics, or treatment), and intervention type (i.e., vaccine, drug, nondrug), so we selected individuals with these affiliations. Where opportunities were identified to increase the depth of understanding of specific considerations – for example, differences between federal and provincial HTA processes, the use of economic methods, and perspectives from decision makers and HTA producers, we sampled additional informants with this expertise (8). The targeted sampling of individuals with health economics training, for example, allowed for deeper exploration of barriers and facilitators of initiatives similar to a disease management approach that have predominantly originated from an economics orientation (e.g., whole disease modeling). Invitations were sent to potential informants via email in four rounds (4 to 6 invitations in each round) between March 2022 and October 2022. Staggering the invitations allowed for assessment of data saturation, revisions to the interview guide, and data analysis to proceed after each round. A sample email, information letter, and consent form are included in the Supplementary material.

All interviews were conducted one to one by a researcher, M.R., with qualitative research training through doctoral degree coursework. Interviews were semistructured using an interview guide shown in Supplementary Table S1. The interview guide evolved as interviews were conducted and analyses were undertaken (8). The interview guide was based on three key domains: conceptualization, value, and implementation of a disease management approach to HTA. During the data collection phase, we initially used the phrase “care pathway approach” because we judged that it would be a more intuitive phrase to support discussion. A care pathway approach was presented to informants as an opportunity to broaden the system of analysis in HTA from one of a single intervention assessment to one that considers the interactions between the full suite of intervention domains (i.e., preventative, screening, diagnostics, treatment, management) that a patient would encounter over the course of a particular disease. To support clarity in the study informant’s interpretation of the approach, we evolved our terminology and definition of a care pathway approach in our interview guide after our first two interviews to read as a “*disease management approach.*” The disease management approach was described as a focus on managing a disease in the context of interventions instead of the traditional approach that aims to manage a single intervention within the context of a disease care pathway. The questions included in the interview guide were discussed with the research team and piloted by documenting anticipated responses and potential probing questions. Interviews were conducted via MS Teams (audio and video) and were transcribed verbatim following the interview. Field notes taken during the interview were added to each transcript and documented as comments and in a separate file (9).

We approached data analysis with a conventional content analysis orientation (10). Three key questions from our interview guide were used as an organizing infrastructure for our data analysis. We used manual line by line coding of the data to identify key themes. The analysis was deepened by reading, re-reading, and writing about the content of the transcripts and discussing findings with the research team (11). Content was revisited repeatedly, and themes identified were further refined in a phase of analysis that was supported by a review of the literature. The identification of relevant literature provided sensitizing concepts that helped frame and further enrich our data analysis (12). A schematic of the analysis process can be found in Supplementary Figure S1.

One author (M.R.) coded the data, and findings were independently verified by a second author (C.M.). The second author coded a subset (4/18) of transcripts to ensure descriptive adequacy, while still allowing for interpretative freedom in the analysis. Findings were discussed (between M.R. and C.M.) to confirm all relevant codes were generated and were descriptively clear. Analyses were completed in Microsoft Word and Excel. We followed the Consolidated criteria for Reporting Qualitative research (COREQ) checklist for reporting which can be found in the Supplementary material (13). Relevant quotes and associated manuscript text were shared with informants for validation.

## Results

Twenty-four invitations were sent out, and eighteen individuals agreed to participate. Six invited individuals did not participate due to a lack of response (n = 3) or lack of time (n = 3). Interviews were conducted between April 2022 and October 2022 and lasted between 30 and 75 minutes in duration. Table 1 describes the key characteristics of participants.

**Table 1.** Characteristics of study participants

	*n* (%)
Female	8 (45)
Professional training	
Clinical training	8 (45)
Formal HTA or economics training	6 (33)
Jurisdiction (primary affiliation)	
Federal	6 (33)
Provincial	12 (77)
Role in HTA (current or former)	
HTA producer (involved in the production of HTA reports and recommendations)	14 (78)
HTA producer and decision maker (involved in the production and use of HTA reports and recommendations)	4 (22)
Appraisal committee experience (member or chair)	4 (22)
Intervention type (primary)	
Vaccines	3 (17)
Drugs	6 (33)
Screening, diagnostics, nondrug treatments	9 (50)

Abbreviation: HTA, health technology assessment.

What follows is a summary of the discourse related to three key questions that were addressed during the interviews: (i) The conceptualization of a care pathway approach to HTA; (ii) The value of a care pathway approach to HTA; (iii) The system capacity to shift to a care pathway approach in HTA.

### Conceptualizing a care pathway approach to HTA

We first capture how informants engaged with the idea of a care pathway approach to HTA by considering what it meant to informants and what sets it apart from other initiatives in the system.

#### How is a care pathway approach interpreted?

Some informants were unclear on the meaning or practical significance of a care pathway approach. There was some variability in how the concept was interpreted; that said, each interpretation was generally understood to have three key features that collectively differ from the conventional HTA process in Canada. First, the care pathway approach involves considering multiple interventions along a care pathway instead of *“…thinking only about one intervention compared to its exact correlate comparator.” (IN02)* Second, a care pathway approach focuses on managing a disease instead of managing intervention(s) in the context of a disease. Informants perceived this approach as *“…looking more holistically at the system and what could be advantageous or beneficial” (IN02)* or framing thinking about the conduct of HTA as, *“…here’s the disease and you can treat the disease in the following ways.” (IN08)* Third, informants generally recognized that the care pathway approach did not simply mean looking at each individual intervention along the disease pathway, but that it considered the relationships between interventions. The relationships between interventions could play out in different ways – communicated by informants as synergies, optimization, trade-offs or choices, sequencing, and interplay, and could impact priorities for innovation as well as how HTA assessments and recommendations are structured. One informant illustrated this feature with an example of the importance of recognizing the relationship between screening and treatment: “*[when you think about] what kind of screening program would be optimal … you cannot disconnect that from the question at the time of who are you going to pay for treatment…?” (IN10).*

#### How does a care pathway approach compare to other initiatives tested or currently in place within HTA?

A care pathway approach to HTA was generally not interpreted by informants as a new idea; however, there was recognition that a single-intervention, technology-centered approach remains the predominant HTA framework in practice. Some informants have had discussions in their organizations about strategically moving in the direction of a broader, system-level approach, or saw their organizations as already implementing elements of a care pathway approach in their HTA assessments. Yet there was recognition by several informants that *“… the HTA framework that is used for some of these … interventions doesn’t maybe go as far as what you’re proposing” (IN02).* The general impression was that there are no systems internationally that take a care pathway approach; *“NICE might be the closest one to thinking about maybe a model of care kind of approach or something” (IN08).* Initiatives such as condition-level reviews or therapeutic reviews at the federal level, or mega-analyses and health evidence reviews at the provincial level were flagged as initiatives that would be considered larger disease-level reviews versus the traditional HTA. Likewise, whole disease modeling and OncoSim models were identified as disease-based decision-analytic modeling approaches; however, none of the initiatives discussed were thought to completely align with what a care pathway approach is intended to achieve.

### Value of a care pathway approach in HTA

We next explored perspectives on the potential value of a care pathway approach. In this section, we discern if the informants interpreted the features of a care pathway approach as ones that would enhance the impact of HTA and if the approach could be used to address HTA or health system challenges.

#### Is there value to a care pathway approach in HTA?

There was some hesitation to affirming or rejecting a care pathway approach to HTA without knowing exactly what it would look like. One informant suggested that the HTA process has worked well for years, indicating that there may not be a need for change. Generally, however, informants engaged with the idea of a potential future state of HTA defined by a care pathway approach and suggested specific features of the approach that, if achieved, could be valuable to the practice of HTA. *“…I can anticipate a future where … we might be interested in trying to balance out the cost effectiveness of the prophylactic intervention compared to treatments, and I think we’re going to get challenged if we’re trying to do that.” (IN02)* A care pathway approach was generally interpreted to provide value if it could meet certain timelines, demonstrate the impact it has on the system as a whole and population health, and considered aspects of diversity, equity, and inclusion. It was clear that there were priority areas (e.g., certain diseases like COVID, or in high-cost diseases) where a care pathway approach would be most relevant. One informant indicated that *“…when we go to psychiatrists who care for people with major depression, they’re all over this work. They don’t want to think about one technology.” (IN14).*

#### How can a care pathway approach be used to address an HTA or health system challenge?

The themes that emerged from informants about the value of the care pathway approach could be interpreted to align with the original triple aim (14) – the pursuit of specific goals to achieve high-value health care: improving population health, enhancing the patient experience, and reducing the per capita cost of care. It was evident that the idea that a care pathway approach could be used to address challenges that the HTA and health system are currently facing resonated with informants. For example, informing discussions around patient choice and suboptimal care: “*I do see value in finding ways for the public and patients to better understand the package of care and the choices.” (IN01),* creating a space where “…*both arms of the government could come together and talk.” (IN10)* and setting up a platform for open dialogue about managing high-cost areas. The care pathway approach could be used to promote a proactive healthcare system. Informants suggested that this approach could enable more effective and efficient decision making across a technology’s lifecycle.

### What is the system capacity to shift to a care pathway approach to HTA?

In this final section, we explored informant’s perspectives of key barriers and facilitators to implementing a care pathway approach in HTA.

#### What are the barriers and facilitators to implementing a care pathway approach in HTA?

Four key themes captured the barriers and facilitators to implementing a care pathway approach to HTA that were highlighted by informants: capacity concerns, data and analytic considerations, system constraints, and political considerations (illustrated in Tables 2–5). We have linked the barriers and facilitators within each of the four themes described as they represented the flip sides of the same phenomenon. It was clear that the barriers to implementing a care pathway approach were not perceived as insurmountable; informants identified opportunities to build on the strengths of the system with this approach.

**Table 2.** Capacity considerations (expertise and education, financial, and comfort with uncertainty)

Subtheme	Barrier or facilitator	Description of theme and subtheme	Illustrative quote(s)
Expertise and education	Barrier	Informants described current challenges in HTA such as a lack of understanding of concepts such as opportunity cost, cost-effectiveness thresholds, and equity and ethical considerations by HTA practitioners and stakeholders. There was an expressed need for individuals with experience in assessing interventions that fall into intervention domains such as prevention, screening, and diagnostics which typically do not receive as much attention in HTA.	*“some of the senior players … don’t really understand the implications that the idea that one decision implicates other people.” (IN09)* *“I just find the HTA world is really struggling to figure out what to do with equity considerations. We live in a world where there’s a lot more accountability and requests for paying attention to indigenous health and health equity and I find the HTA processes are really they’re not designed to deal with that” (IN06)* *“… that’s a really tricky ethical question and it’s probably also a very hard legal question, whereas like some of our HTAs are not.” (IN10)*
	Facilitator	Some informants had suggestions to provide more education around opportunity cost. Others emphasized that the appropriate expertise would be needed to assess and appraise the evidence as part of review teams and committee members.	*“I think you know we need to have a much better education about what the implications of decisions are…we need to be able to have that embedded in decision making and then the concept of care pathways becomes clearer because if we think about the yeah, if we make one decision implicates other people, then the concept that we make one decision implicates other decisions becomes very, very obvious” (IN09)* *“So you need a committee then that can either review that kind of evidence or understand that kind of evidence and then deliberate on that kind of evidence as well.” (IN08)*
Financial	Barrier	Informants described a key barrier related to funding and incentives for looking at a care pathway approach – no particular decision maker is asking for a full care pathway approach assessment. There are also pressures to make more efficient use of the dollars going to HTA assessments – doing more within the current funding envelope.	*“It’s about grasping like bits of funding from one place and from another place. You know, it’s not about like someone coming and saying like you know, we want to improve care pathways for people who have a diagnosis of major depression” (IN14)*
	Facilitator	There was recognition that the reimbursement and granting environment currently incentivizes pharmaceutical companies and treatment interventions and that there could be opportunities to look at the implications of these. Peer-reviewed public funding could be a source of groundwork for developing care pathway considerations in HTA – specifically for economic models. Potential role at federal level for economic models to support provinces because this seems to be where the resource concerns are.	*“right now we incentivize pharma, who runs almost all of the clinical trials, and I guess what they’re interested in selling, right? It’s only drug. And so they compared drug to non drug or drug to active drug and they’re not thinking about backing up the system. They’re not in that space. It comes back to where the funding bucket is right” (IN08)*
Comfort with uncertainty	Barrier	From those informants who have experience with broader HTA questions, the uncertainty that comes with these assessments was highlighted. Concerns about HTA practitioner comfort with uncertainty and writing policy discussions that are not typically conducted within traditional HTA. Further, how broad do you draw the boundaries of the scope of analysis?	*“It’s just a mess. And I don’t mean mess in a bad way, it’s just that it’s just, you know, it’s tangled. It’s complicated. And you don’t get as clear as an answer, and some people are less comfortable with that kind of uncertainty”(IN13)*
	Facilitator	Although a care pathway approach is expected to increase the uncertainty, there were recommendations for identifying ways to highlight the key implementation recommendations. The care pathway approach could also provide a lens to better demonstrate how the system could benefit and start a patient-centered conversation.	*“HTA documents they produced were like 400 pages long…nobody seems to have a problem with that size of document or that level of comprehensive assessment…it’s just about can you see the wood from the trees with all of that at the end of the day? And whether you’re going to be able to make meaningful recommendations that people can implement” (IN12)*

*Note*: The table describes the theme, subthemes, and provides illustrative quotes.

Informants identified capacity concerns (Table 2) as a key consideration for the implementation of a care pathway approach to HTA. This idea included ensuring that appropriate expertise and education are in place both at the assessment (reviewer and analytical expertise) and appraisal level (committee expertise), ensuring there are sufficient financial resources, and that there is an appetite or skill set to embrace uncertainty. Existing HTA concepts that factor into understanding a care pathway approach such as opportunity cost, and equity and ethical considerations were described by informants as commonly misunderstood. There was recognition, however, that some concepts have the potential to become clearer with the implementation of a care pathway approach.

Data and analytic considerations (Table 3) were particularly concerning for informants with the implementation of a care pathway approach to HTA. There was recognition that provinces are information-rich and making use of data available at this level of the system would be important. The complexity of the analytic task and the limited experience that HTA has in reviewing medical devices and clinical interventions were also highlighted as potential challenges.

**Table 3.** Data and analytic considerations (data availability, experience with medical devices and clinical interventions, complexity)

Subtheme	Barrier or facilitator	Description of theme	Illustrative quote(s)
Data availability	Barrier	Informants described potential concerns with data availability – both clinical and economic to inform decision making. There was concern that data is already a challenge in HTA – including just understanding standard of care and the natural history of disease. It is not how the clinical trials are designed and do you have data to create linkages between each of the intervention domains. Further, it was acknowledged that some disease areas have more data than others.	*“I don’t think that people who choose the clinical trial outcomes sit down and take the approach that you’re thinking of when they choose it. It just happens to be in some areas that we would see a clinical trial outcome that would give some insight into the pathway.” (IN06)*
	Facilitator	Despite clinical trials not providing the appropriate evidence, there was recognition that the provinces have the data available. So, the care pathway approach would be best implemented at the provincial level. But need to balance resource capacity at the provincial level with specificity of the model – could CADTH offer tailored models? There was also a recommendation by one informant to set up data according to International Classification of Disease (ICF) coding to allow for the linkage between trial and administrative data.	*“one of the things that we’ve been blessed with is actually having access to provincial data. And, you know, that’s a distinct Canadian advantage, I think, is just the data availability” (IN14)* *“if we organize the whole of health care system and all of our data gathering and all of our clinical trials around The WHO ICF (International classification of function)… as a way of organizing everything that we think about in healthcare, then it wouldn’t be complicated or costly or timely to pull it back together at the end…because from the beginning we would have used meaningful outcome measures in the trials.” (IN06)*
Experience with medical devices and clinical interventions	Barrier	Compared to pharmaceutical products, medical devices and clinical interventions are a rapidly evolving area of innovation without a clear HTA pathway, and outcomes/assessment processes that are less clear.	*“it’s very challenging in a prophylactic space with something like vaccines, which are to be in most cases used incredibly broadly for healthy populations.”(IN02)* *“it’s not an easy process to connect up with the right areas of the ministry, and then it’s very hard for them to commit in terms of budgeting.”* *(IN03)* *“so there’s a lot of evolution, rapidly more rapidly than in the drug area.”* *(IN07)*
	Facilitator	One informant mentioned that the more you look at certain outcomes or technologies, the better quality they will become. A care pathway approach allows medical devices and clinical interventions to be featured where they may otherwise not have been. Informants talked about opportunities to build on what the system is primed for – infectious disease and mental health.	*“I think it is important though that if there is a prevention that has a huge impact on the use of a particular drug or the need for a particular drug, I would suggest it probably could be included in some of our reports.” (IN17)*
Complexity	Barrier	Informants described the complexity of the analytic task of trying to pull all of the pieces together with a disease management approach. There is never just one care pathway.	*“And they are certainly more complex. And I don’t just mean that in the in the methods, the sense I mean that in the way you have to engage with different stakeholder groups and collect and develop evidence and understand the evidence. “(IN13)* *“So it’s more of that holistic sort of a view in terms of trying to work through that, which then I think requires more of those complex program analysis and decisions. At least at the recommendation level. Operationalizing that then adds a bit more complexity on to that one also” (IN04)* *“it’s pretty complicated. I guess I’m. I’m having difficulty seeing how you piece it altogether.” (IN11)*
	Facilitator	Facilitators to manage the complexity of a disease management approach included a recognition that it will not be a reasonable approach for every technology that comes through and that there would likely be some benefit in prioritizing certain areas that are likely going to be particularly challenging to decision makers – such as rare diseases, cancer, pediatrics.	*“That’s not reasonable approach every time. And I think it isn’t reasonable every time because you’ve got limited resources” (IN09)* *“Rare diseases, cancer, or Pediatrics as well would be. The different set of issues slightly with Pediatrics but. You know, when I talked to the leadership at CADTH and the people who hold the budgets from provincial level is the rare disease is one that they’re really worried about at the moment; They’re the ones I would start with because I think that’s where we’ve got our biggest challenges in terms of allocating resources.” (IN12)*

*Note*: The table describes the theme, subthemes, and provides illustrative quotes.

System constraints (Table 4) generally shared a common concern about the siloed nature of the healthcare system – from budgetary, program, and organizational silos, to silos between pharmaceuticals and nonpharmaceutical interventions, public health and the healthcare system, and health and nonhealth sectors. Informants described examples of the HTA community and organizations starting to bridge the gaps between silos, and that some of these silos – such as integration of a vaccine and drug recommendation – would be best accomplished with HTA at the provincial level.

**Table 4.** System considerations (budgetary and program silos, organizational silos – HTA assessment processes, silos between pharmaceuticals and nonpharmaceutical interventions and their delivery, and silos between public health and the rest of the health system and health and nonhealth sectors)

Subtheme	Potential barrier or facilitator	Description of theme	Illustrative quote(s)
Budgetary and program silos	Barrier	Informants described the challenges of separate budgets and programs within a Ministry department. If there was evidence to suggest a potential reallocation of money between budgets or if there was a need to synthesize evidence between two programs, this would be challenging due to competing demands within a given budget, legislative barriers, and different approval processes. This challenge would be exacerbated with a care pathway approach.	*“I mean part of the problem is well, you think that’s a great idea, but then the drug budget isn’t the same as the screening budget.” (IN09)* *“even if they transfer cheaper, even if they transferred me some of the money. You know, they actually have a pile of 20 things that they’re not funding, so it’s not like there’s, like, green money, really like green dollars really laying around….people, talk about silos and it’s not really silos. It’s lack of understanding of the competing demands for any decision.” (IN01)*
	Facilitator	Informants describe the importance of having people at the table at the start of discussions and the value that HTA could bring in convening various groups. There was also recognition that some provinces are set up in disease-focused environments.	*“you have to build a coalition of the right people at the outset. So if you pick a disease area you need to have the right people around the table from the get go.” (IN12)*
Organizational silos – HTA assessment processes	Barrier	Informants described the silos in the HTA processes both between and within organizations – vaccines are assessed and appraised separately from drugs by different organizations. Within organizations, drugs and nondrugs are typically assessed separately at the federal and provincial levels. However, there is variation at the provincial level. One province, Quebec, has greater integration of drug and nondrug interventions.	*“But there tends to be this siloed system, and it is siloed … in terms of the advisory bodies as well.” (IN02)*
	Facilitator	There was a sense of movement within organizations for greater integration between silos – driven by questions that were being asked and the recognized value of doing so. Informants also highlighted potential ways that a care pathway approach could move forward within the HTA process that would build on the strengths of the system (e.g., setting up an algorithm at CADTH to identify cases).	*“I know they’ve transitioned like purposely. Intentionally transitioned away from the single technology HTA and more looking at care pathways.” (IN15)* *“that’s why they now have an HTA committee and not just a drug committee that we need to take a wider view of the decisions that we make.” (IN09)*
Silos between pharmaceuticals and nonpharmaceutical interventions and their delivery	Barrier	Informants described a recognized lack of consideration of nonpharmaceutical alternatives within assessments of pharmaceuticals and uncertainty in how that came to be. This system-level consideration would be particularly challenging for implementing a care pathway approach.	*“…this is a drug and it’s going to fall under the drug budget and may only comparators that I could have are the drug budget comparators.” (IN08)* *“This strange distinction between things. Things have to be different for drugs versus non-drugs. Which obviously does not fit with a with a sort of disease pathway sort of approach.” (IN14)*
	Facilitator	Similar to above, there was a sense of movement within organizations for greater integration between drug and nondrug and building with some ideas for implementing a care pathway approach that would build on the strengths of the system.	*“So I still think that the appraisal of individual drugs is going to be necessary because yeah, I mean like these things or individual technologies, these things are being developed and thrown at the health systems, you know, left, right and center.” (IN14)*
Silos between public health and the rest of the health system and health and nonhealth sectors.	Barrier	Informants described a particular siloing between public health/prevention and the rest of the healthcare system – at the clinician level, provincial level, and federal level.	*“my experience of you know, the prevention and the treatment people talking across my career, is not amazing.” (IN13)*
	Facilitator	Generally, informants believed that bringing together public health and the rest of the health system could best be accomplished at the provincial level for the right kind of questions and that it does not necessarily have to be a formal relationship.	*“horizontal groups could be used for that. We probably will have to do something that’s a bit more informal. I would say making linkages, those sorts of things.” (IN04)*

*Note*: The table describes the theme, subthemes, and provides illustrative quotes.

Political considerations (Table 5) highlighted by informants included the pressures on decision makers, incentives for change, and the multistakeholder HTA environment. Appropriately managing political pressures could include respecting provincial and territorial autonomy, identifying priority areas, and ensuring that the approach is “*thoughtfully characterized*” (IN01). Other constraints expressed by informants were not unique to the integration of a care pathway approach – reflecting common constraints on change agendas in HTA or other environments.

**Table 5.** Political considerations (political pressures, change management, multistakeholder environment)

Subtheme	Potential Barrier or Facilitator	Description of theme	Illustrative quote(s)
Political pressures	Barrier	Informants described the political pressures facing decision makers – making evidence-based decision-making challenging.	*“…so clearly we’ve got a huge amount of political barriers” (IN09)* *“You know, perhaps going to say we can’t afford it so you know, they might not want to publish the findings. And so you have to deal with different issues like that.” (IN13)* *“…except that after that there are a lot of pressures. You get the data with the economic indices and then you have the lobbying. Lobbying from the drug companies, lobbying for the vaccine companies, lobbying from the pediatricians internal GP’s nurses. The media. So the final decision will be influenced by those contextual external influences.” (IN05)*
	Facilitator	Informants described facilitators to the political pressures including respecting provincial and territorial autonomy, identifying priority areas, and ensure that the approach is thoughtfully characterized.	*“We do need to respect that the provinces and territories are responsible for their own health systems and have to make decisions, and the fiscal situation in different provinces is quite different at times.” (IN12)* *“And again for patients and again, government decision makers have to manage the politics of it. The media of it, and that, you know, if there isn’t a way for that to be thoughtfully characterized, it would be a difficult sell even if they loved it.” (IN01)*
Change management	Barrier	Informants shared that change is hard at all levels of the system – from the general belief that the Canadian system is not adaptable, to challenges with changing clinician behavior and in changing the methods used by scientific disciplines.	*“You will get some entrenched people there who want to defend their territory at all costs.” (IN12)* *“…once you have an established field, it’s hard to get people to change what they do.” (IN13)*
	Facilitator	Several potential facilitators were suggested by informants including someone to take ownership of the initiative, providing demonstration projects, motivating senior leaders as well as those on the ground, and a recognition that change is gradual and may not be a perfect solution, but it is a step in the right direction.	*“…highlight what the things we are losing out on because of the system and so that you know, if the system could adapt, this is how we could benefit” (IN09)* *“…it’s a huge leap forward I feel from the technology focused work and that. But yeah, I mean we shouldn’t be naive to think that there’s not a further leap that might be needed or will be needed at some point.” (IN14)*
Multistakeholder environment	Barrier	Informants described the challenges of multistakeholder environments and that it is unlikely that you are going to make everyone happy in every decision.	*“Unfortunately, it’s quite hard because you need to motivate senior leaders as well as some people on the ground. Multi stakeholder environments are just difficult.” (IN12)*
	Facilitator	Facilitators of achieving success for a care pathway approach in a multistakeholder environment are having the decision makers at the table from the outset, understanding and aligning with government budget cycles. There was also recognition that Canada is a very inclusive and respectful environment for building these types of collaborations.	*“…you have to build a coalition of the right people at the outset. So if you pick a disease area you need to have the right people around the table from the get go.” (IN12)*

*Note*: The table describes the theme, subthemes, and provides illustrative quotes.

## Discussion

A care pathway approach to HTA is a disease-based, multi-interventional, and dynamic approach to supporting evidence-based healthcare decision making. Informants generally saw value in this approach as a methodological and structural advancement – that is, generating evidence that supports consideration of intervention options across the disease trajectory, and creating opportunities for system level deliberation. A sentiment emerged that a care pathway approach may not be needed every time, and selecting the most impactful cases would be important. There are opportunities for a care pathway approach to be implemented as a complement to the current approach without major restructuring of the HTA system – ones that build on the strengths of the existing system in Canada and involve a shared responsibility and ownership between federal organizations and provincial decision makers to balance resource constraints, efficiencies, and relevance.

A care pathway approach was not necessarily interpreted by informants as a new idea – discussions regarding this concept have occurred at the pan-Canadian level (CADTH) and similar types of thinking have taken place at the provincial level with mega-analyses in Ontario, health evidence reviews in Alberta, and as a standard practice for all reviews in Quebec (i.e., a drug submission offers the opportunity to ask what is the best option for the health system). Although these initiatives emulate the idea of thinking about the decision problem in HTA as going beyond the traditional mold of assessing a single health technology, the current HTA framework remains predominantly technology centered. A care pathway approach requires a shift in thinking to have the focal point of an HTA as the disease and not the technology. Three key features collectively distinguished a care pathway approach to HTA from the conventional HTA approach in Canada: disease-based, multi-interventional, and dynamic. We compare a care pathway approach to other initiatives, ideas, and methods that were mentioned by informants in Supplementary Table S2. Other approaches to HTA, such as Optimal Use reports at CADTH and pathway approaches at Cancer Care Ontario (CCO), that were not mentioned by informants may also be interpreted as similar to our work. These approaches may offer more comprehensive assessments; however, our interpretation is that optimal use reports are still generally focused on single domain of interventions, and pathway approaches at CCO are closer to reflecting clinical guidelines which are not intended to optimize the care pathway at the population level. A “disease management approach” to HTA was thought to better reflect the intent of what, in our interviews, we initially defined as a “care pathway approach.” We did not endeavor to do an in-depth comparison between a disease management approach and other initiatives; however, future research could comprehensively assess the similarities and differences between HTA process initiatives.

One could argue that over the past decade, HTA has been experiencing a paradigm shift. The idea of “living” HTAs, lifecycle assessments, and health technology management continue to be primary considerations in HTA discourse. Several informants in our study highlighted the parallels between a disease management approach and these more mainstream HTA paradigms. For example, one informant saw a disease management approaching fitting in with the idea of health technology management, or that a disease management approach could be deployed as an approach to manage suboptimal use of health interventions. We view these parallels as a strength of a disease management approach. A disease management approach could be used to operationalize a health technology management strategy in HTA by creating an inherent need to reassess technologies each time a disease-based review is undertaken.

The barriers and facilitators to implementing a disease management approach were similar to those that have previously been identified for other proposed “change” agendas in HTA. For example, Hopman et al. (15) identified accessibility (availability and knowledge) and acceptability (scientific/technical, structural/institutional, and ethical and political) barriers to including economic evaluation in immunization policy making. Findings from Clausen et al. (16) highlighted the need for a cultural shift, improved data infrastructure, committed investment, and increased collaboration to optimize the implementation of real-world evidence in cancer drug funding decisions in Canada. Stojanovic et al. (17) identified data, conflicting stakeholder priorities, and appropriate methodology as common challenges. At the broadest level, the ISPOR-identified top 10 challenges (18) in HTA overlap with the barriers we identified. These findings suggest that although there are likely barriers to the implementation of a disease management approach to HTA, the field will continue to grapple with these challenges regardless of any proposed change agenda.

We anticipate that creating platforms for assessments at the disease level could facilitate opportunities for early-phase horizon scanning initiatives, optimization between typically separate intervention domains (i.e., primary prevention, screening, diagnostics, and treatment), and for considerations of “what-if” scenarios in the context of future innovations. The platform would also have the potential to continue to advance patient and public engagement in HTA by creating opportunities for elicitation of patient preferences across intervention domains, facilitating greater public involvement, and creating long-run assessment efficiencies and expertise. Public health and preventative interventions are typically underrepresented in HTA analyses (4;17), so a disease management approach could allow for greater traction for these and other typically underassessed intervention types. These opportunities are anticipated to contribute to a more proactive system in Canada and align with other calls for HTA reform (19), It is likely that if a disease management approach to HTA were to be established in the current system, existing HTA prioritization processes and tools (20–22), patient, clinician, and guideline groups that are predominantly disease-focused, and disease classification systems within data holding systems could be used to help identify the most relevant and impactful cases.

## Limitations

There are several limitations to our study. First, many informants struggled to gain clarity about the idea of a “care pathway approach” to HTA, in some cases identifying the approach as inherent in the HTA framework. The care pathway approach has not previously been conceptualized and it was our intent to be open to informant’s interpretation of the approach, stimulate discussion of comparisons to other initiatives, and to share thoughts on how the approach could be implemented in practice. Despite some misinterpretation of the distinguishing features of the care pathway approach, informants were able to constructively identify its elements and impact. Second, there may be other ways of conceptualizing the decision problem in HTA beyond that of disease management. Arguably, a disease management approach to HTA could still be considered technology centric. In selecting a disease-level system of analysis, we believed that this would be an incremental and feasible step for HTA to shift the focal point of assessments to be one that at a macrolevel is more patient-centered and more aligned with a broader health system perspective compared to a predominantly technology-focused, patient-informed approach. Third, our sample population consisted primarily of individuals working as HTA “producers” (federally or provincially) and primarily in the diagnostic and treatment space. We sought to include individuals who were in decision-making roles and those whose work focuses on primary prevention; however, representatives with this expertise were few. Further exploration of a disease management concept with these individuals would be valuable. Additionally, we specifically targeted the inclusion of some individuals who had professional training in health economics and individuals who had experience working in jurisdictions that have sought to broaden the evaluative space in HTA assessments (e.g., British Columbia, Alberta, and Quebec). This focus may have led to overly optimistic views of the value of a disease management approach to HTA. We balanced these perspectives in our results by highlighting areas where skepticism of the disease management approach was apparent. Fourth, interviews, transcription, and analysis were performed by a single reviewer. This may have an impact on the trustworthiness of our results; however, research suggests that this approach is reasonable especially when using inductive content analysis (23). We mitigated this potential limitation by having a second reviewer (C.M) code a subset of transcripts and confirm the appropriateness of key themes and subthemes identified. Furthermore, our research team had several meetings as data analysis proceeded to ensure the rigor of the analysis. This included achieving consensus on the key question sets that framed the foundation for the analysis and that the analysis trail was clearly documented and substantiated by informant quotes. Lastly, our research did not set out to explore the value of a disease management approach to other system stakeholders such as local or hospital-based HTA organizations, patient representatives, the public, manufacturers, or private payers. It was our intent to focus on HTA system capacity and understand the constraints to the assessment and appraisal process for federal/provincial recommendations and decision making; however, additional perspectives on this approach is an important contribution in future work.

## Strengths

Our research has several strengths. In our analysis, we drew on existing literature to frame the barriers and facilitators to implementing a disease management approach to HTA. Further, we offered a unique framing of these findings in linking the potential barriers with potential facilitators. Second, our sample population offered wide-ranging and insightful comments that highlighted potential optimism and skepticism of a disease management approach to HTA. Third, we implemented a rigorous analytical process to derive key themes and subthemes that resonated with those found in existing literature. Lastly, we sought to ensure credibility, transferability, dependability, and confirmability in our research (24). Credibility was established by continuous revisiting of transcript data and comparing it to themes, continuously comparing ideas and insights between transcripts, linking results with existing literature, and sharing the draft results with informants. We aimed to facilitate transferability of findings by describing the contextual conditions associated with the data to the extent possible. We discussed, documented, and illustrated our approach to analysis continuously within our research team and by sharing this process transparently within the manuscript to ensure dependability. Lastly, we documented and reflected on potential biases before, during, and after each interview as well as engaged in continual reflexivity during analysis to ensure confirmability.

## Conclusions

HTA has a strong presence in the Canadian healthcare system, and efforts to play to its strengths and shift with the fluid needs of the system are important for delivering appropriate evidence to decision makers. Our research aimed to inform the consideration of an HTA process change, a disease management approach to HTA, that could be considered as a complimentary approach to existing processes to better inform a proactive, resilient, and sustainable healthcare system in Canada. Moving from intervention management to disease management is one option for a purposeful systems-thinking approach to enhancing the impact of HTA.

## Supporting information

Richardson et al. supplementary material 1 Richardson et al. supplementary material

Richardson et al. supplementary material 2 Richardson et al. supplementary material

## Supplementary material

The supplementary material for this article can be found at https://doi.org/10.1017/S0266462323002696.
