# Supplementary material for: Moving from intervention management to disease management: a qualitative study exploring a systems approach to health technology assessment in Canada
Source: Int J Technol Assess Health Care. 2023 Nov 6;39(1):e67. doi: 10.1017/S0266462323002696 (PMC11579670; doi:10.1017/S0266462323002696)
Supplement: Richardson et al. supplementary material 2 — Richardson et al. supplementary material [file S0266462323002696sup002.docx]

# Supplementary File

Contents

[Supplementary File 1](#_Toc142814589)

[Draft Email Requestion Participation 2](#_Toc142814590)

[Information Letter 3](#_Toc142814591)

[Consent Form 6](#_Toc142814592)

[Supplementary Table 1: Interview guide support during interview: domains, key questions, and potential probes 8](#_Toc142814593)

[Supplementary Figure 1: Summary of Analysis Process 11](#_Toc142814594)

[Supplementary Table 2: Comparison of existing initiatives to a care pathway approach to HTA 12](#_Toc142814595)

[References 20](#_Toc142814596)

## Draft Email Requestion Participation

EMAIL REQUESTING PARTICIPATION: Sent via: [marina.richardson@mail.utoronto.ca](mailto:marina.richardson@mail.utoronto.ca)

Dear _________________,

I am reaching out to see if you are available to participate in a qualitative research study that is exploring the potential value of using a care-pathway approach to assessing evidence and making decisions about recommending or paying for health care interventions (e.g., drugs, medical devices, screening interventions, vaccines, etc.) from a health technology assessment (HTA) perspective. This research is part of my PhD dissertation through the University of Toronto (Institute of Health Policy, Management, and Evaluation) supervised by Dr. Beate Sander.

Your involvement is expected to consist of an approximately 30-to-60-minute interview over video or telephone. A follow-up conversation may be requested for clarification purposes and this will be kept to no more than 30min. No preparation is required.

I have attached an information letter that contains additional information, conditions for participating, potential risks and benefits, and confidentiality, as well as a consent form for your review.

Please let me know if this is of interest and I will be in touch with next steps.

Thank you for your consideration,

Marina Richardson

[Marina.richardson@mail.utoronto.ca](mailto:Marina.richardson@mail.utoronto.ca)

## Information Letter

**Information Letter**

*Exploring opportunities to integrate care pathway approaches in HTA*

**INTRODUCTORY INFORMATION**

Thank you for considering being involved in our research study.

Our research study seeks to explore the potential opportunity of using a care pathway approach to assessing evidence and making decisions about recommending or paying for health care interventions (e.g., drugs, medical devices, screening interventions, vaccines, etc.) from a health technology assessment (HTA) perspective using qualitative research methodology. This research is part of my (Marina Richardson’s) PhD dissertation through the University of Toronto (Institute of Health Policy, Management, and Evaluation) supervised by Dr. Beate Sander. Funding to support this project has been provided by a Canadian Institute of Health Research (CIHR) Doctoral Research Award. Ethics approval has been received by the Health Sciences Research Ethics Board at the University of Toronto.

**who are we seeking involvement from?**

Our study is seeking individuals who have knowledge and experience in the field of HTA. This could include individuals with an understanding of HTA in Canada (for drugs, medical devices, and/or vaccines), an understanding of the decision-making process, and a working knowledge of HTA methodology with an emphasis on economic methods. Participants may include individuals from, for example, national or provincial HTA agencies (CADTH, National Advisory Committee on Immunization [NACI], Institut national d'excellence en santé et services sociaux [INESSS], Ontario Health (Quality), or related organizations), and individuals involved in decision-making across the disease care pathway – i.e., prevention (vaccines, screening) and treatment (drug and non-drug), and across the decision-making pathway – i.e., HTA organizations, expert committee members, and decision-makers (e.g., hospital administrators, long-term care home administrators, etc.). Our sample size is estimated to be approximately 15 to 20 individuals to sufficiently capture the perspectives across the decision-making pathway (i.e., HTA producers and users) and across the care pathway (i.e., prevention and treatment).

**Why are we seeking your involvement?**

Your experience and involvement in HTA and the decision-making pathway for health interventions is anticipated to provide insight and perspectives as to the potential opportunity (e.g., where in the system and in what form) of a care pathway approach to HTA and economic evaluation in Canada.

**CONDITIONS FOR PARTICIPATING**

Participation in this study is voluntary, and you may refuse to participate, withdraw at any time, and decline to answer any question or participate in any parts of the study with no consequences. If you request to withdraw from the study after the interview and prior to data analysis, your data will not be included in the results. However, if you withdraw after the research team has analyzed your data, your data will be retained, however, no direct quotes will be used. You can no longer withdraw from the study after the data are synthesized. Your participation would involve an approximately 30-to-60-minute semi-structured interview conducted through Zoom or MS Teams web-conferencing. A password enabled meeting invitation will be sent individually to each participant. Voice-only interviews will be accommodated if preferred. With your consent, the interview will be recorded using the record function on Zoom or MS Teams to allow for more details notes and verification of data. Interview recordings will be stored in password protected files on a local computer (separate from participant list) and labeled with the participant ID. We will also ask for your consent to use direct quotes. Refusal to allow for recording of the interview or direct quotes being used will not preclude participation in the study. If consent for recording is not granted, detailed notes will be taken. With your permission, a follow-up call may be requested for the purposes of clarifying points.

No honorarium will be provided. The only anticipated cost to participating is your time.

**POTENTIAL RISKS AND BENEFITS**

There are no perceived risks to participating.

It is anticipated that you will benefit from the opportunity to think critically and creatively about the infrastructure for HTA in Canada. Participation is also likely to allow for a discussion about how to make research and research results fit for purpose (i.e., how to design research that can support decision-makers needs) and may result in capacity-building for participants who are not as familiar with the methods and tools of economic evaluation.

For the scientific/scholarly community, exploring how HTA experts perceive the opportunity of considering the full care pathway in HTA is anticipated to help explain why single technology assessments at single decision points along a care pathway predominate in practice. If modelling a full care pathway is deemed to have potential value in HTA, our research is expected to help provide insight into the development and/or refinement of current HTA methods. Finally, our research is anticipated to set in motion a dialogue within the HTA community on the potential barriers or facilitators to effective collaboration across HTA and health system boundaries to mirror the patient journey along a continuum of care.

**CONFIDENTIALITY**

Interview data that includes personal information (i.e., participant list, consent forms, and interview recordings), will be stored in a password protected folder on a local computer. The participant list will include an identification number for each participant that will be used to de-identify transcripts and field notes, and this list will be kept in a separate password protected file on a local computer. Written field notes or other interviews notes will be immediately transferred to electronic files, and the written notes will be shredded. Data that needs to be shared for the purposes of data analysis (i.e., transcriptions and field notes) will be de-identified and shared via the University of Toronto OneDrive. All data will be stored for 7 years and then destroyed.

**Publication of results**

It is our intent to disseminate the findings of this research in peer-reviewed publications and through public presentations at research conferences. In our publications, no personal identifiers will be reported. In the case of direct quotes being reported, a naming convention of IN1, IN2, etc. will be used and only quotes that do not risk this anonymity will be reported. Identifiable information (for example, name and role) may be asked during the interview; however, this information is intended to be used for contextual information.

**RELEVANT CONTACT INFORMATION**

The research team is available for contact by the participants with questions or comments at any time:

- Marina Richardson, PhD Student, University of Toronto, Email: [marina.richardson@mail.utoronto.ca](mailto:marina.richardson@mail.utoronto.ca)
- Beate Sander, Associate Professor & Faculty Co-Lead Health Technology Assessment program, Institute of Health Policy, Management and Evaluation (IHPME), University of Toronto

Phone: 416-634-8020; Email: [beate.sander@uhnresearch.ca](mailto:beate.sander@uhnresearch.ca)

We also welcome participants to contact the Research Oversight and Compliance Office – Human Research Ethics Program at [ethics.review@utoronto.ca](mailto:ethics.review@utoronto.ca) or 416-946-3273 if they have questions about their rights as participants at any time.

## Consent Form

**Consent Form**

*Exploring opportunities to integrate care pathway approaches in HTA*

I have read and understood the study information letter for the research study “*Exploring opportunities to integrate care pathway approaches in HTA”.*

Yes  No

I voluntarily consent to be a participant in this study.

Yes  No

I understand that I can refuse to answer questions and I can withdraw from the study at any time, without having to give a reason. If I request to withdraw from the study after the interview and prior to data analysis, my data will not be included in the results. However, if I withdraw after the research team has analyzed my data, my data will be retained, however, no direct quotes will be used. I can no longer withdraw from the study after the data are synthesized.

Yes  No

I understand that information I provide may be reported in the published literature or conference publications or presentations.

Yes  No

I consent to the recording of the interview using the record function on Zoom or MS Teams and that the interview recordings will be stored in password protected files on a local computer (separate from participant list) and labeled with the participant ID.

Yes  No

I consent to direct quotes being used if relevant. All quotes will remain anonymous.

Yes  No

**Signature**

_____________________________ _______________________

Name of participant Signature and Date

**RELEVANT CONTACT INFORMATION**

The research team is available for contact by the participants with questions or comments at any time:

- Marina Richardson, PhD Student, University of Toronto, Email: [marina.richardson@mail.utoronto.ca](mailto:marina.richardson@mail.utoronto.ca)
- Beate Sander, Associate Professor & Faculty Co-Lead Health Technology Assessment program, Institute of Health Policy, Management and Evaluation (IHPME), University of Toronto

Phone: 416-634-8020; Email: [beate.sander@uhnresearch.ca](mailto:beate.sander@uhnresearch.ca)

We also welcome participants to contact the Research Oversight and Compliance Office – Human Research Ethics Program at [ethics.review@utoronto.ca](mailto:ethics.review@utoronto.ca) or 416-946-3273 if they have questions about their rights as participants at any time.

## Supplementary Table 1: Interview guide support during interview: domains, key questions, and potential probes

| **Domain** | **Speaking Points/Key Question** | **Potential probes** |
| --- | --- | --- |
| **Introduction**  What is your role, how did you get into this role? | 1. Could you tell me about how you found yourself into your current role? 2. How your involvement/role in HTA has evolved over the years? | Any observations in the evolution of HTA over the years? |
| **Current Status of a Care Pathway Approach**  How is the rest of the system considered?  Has it come up, is there interest? Are you doing it?  Trying to get at if there is an awareness of this type of approach to structuring the decision problem in HTA? | 1. When I say “care pathway” there seem to be variable interpretations of what I mean by that.    1. To some it may mean huge changes to the way we do our HTAs – for example, doing a detailed review of every single health intervention available in a single disease space – to the other extreme of well, we already incorporate a care pathway approach in our assessment because that’s the essence of an economic model – modeling out the trajectory of the patient with a particular disease.    2. My dissertation is intending a care pathway approach to be somewhere in between with a focus on how different technologies (e.g., drug and non-drug treatment options, or prevention and treatment options) interact within the care pathway. 2. How you define the boundaries of the interventions you consider in your assessment – i.e., non-drugs vs. other non-drug interventions? I’m trying to get an understanding of how comprehensive your assessment is of the overall disease landscape? 3. Why do we construct the decision problem in the way we do – i.e., individual technologies? 4. Have you considered other ways that the scope of an HTA assessment could be conceptualized? 5. Did you or have you seen any shifts in the system towards pathway/disease management?    1. Can you talk about if or how this type of approach or idea is being discussed? | **Formally**   - Have you requested this, or are you interested in this? - Have funders, decision-makers ever requested this? - Do you ever consider how changes to upstream options might impact the treatment intervention you are assessing?   **Informally**   - What do conversations **within your organization** look like when it comes to care pathway considerations that involve HTAs for interventions that are not typically conducted by your organization? - Do you have conversations with individuals in **other organizations or other parts of the** care pathway when you are doing your assessments? |
| **Capacity**  Is there capacity for this (internally and externally)?  Are any structural constraints coming to mind? Anything outside of your organization that influences your capacity to do so?  Any analogous considerations? | 1. As you can imagine, the “care-pathway approach” could be operationalized in many ways throughout the HTA process (e.g., scoping, assessment, appraisal, implementation, monitoring), or within different value dimensions of HTA (e.g., patient and caregiver perspectives, clinical evidence, economic evidence, implementation considerations, etc.). This is something that I would like to explore within the interviews – both how the dynamics of “other interventions” are considered currently within HTA, and if there are any opportunities (value and capacity) to do so in HTA moving forward. 2. If someone did ask your team/analysts to produce or use an HTA assessment that considers multiple interventions along the care pathway (i.e., so not just vaccine X or treatment Y or policy Z), but also how other upstream/downstream options compare or influence other interventions along the care pathway, do you think you could?    1. What would it look like?    2. Would anything need to change in the system? 3. Are there ways we could get around the funding envelopes? 4. Whose responsibility would it be to produce? | - What might be some of the structural constraints that would prevent this type of care pathway approach from being used in HTA? - What might be some structural considerations that would encourage the use of this type of approach? - What type of collaboration would this require? - If all health care budgets were under the same roof, how might that change what information is requested and what types of decisions are made? - If it is already being done, what are the opportunities and challenges that it presents for the organization, committees, decision-makers. |
| **Value**  Do you think considering other parts of the system would be valuable? | 1. Do you think this is the direction the system needs to go? 2. If we take a step back and think about the system as a whole – what are your thoughts on a care pathway approach? Would the system benefit from it? Are there too many hurdles? What are some of these constraints? 3. What are the upsides and downsides? 4. Are there other pressing system-level priorities? | - Can you provide an example of a time when a care pathway approach to HTA may have been a useful approach to addressing a decision-makers request/problem? (Economic or non-economic component of HTA) - Any other examples? |
| **Concluding thoughts**  Anything else that you’d like to mention? | 1. What do you think about what we’ve talked about? 2. Parting thoughts? About prospects of care-pathway approach in HTA? | - What would have to change to have it function this way? |

## Supplementary Figure 1: Summary of Analysis Process


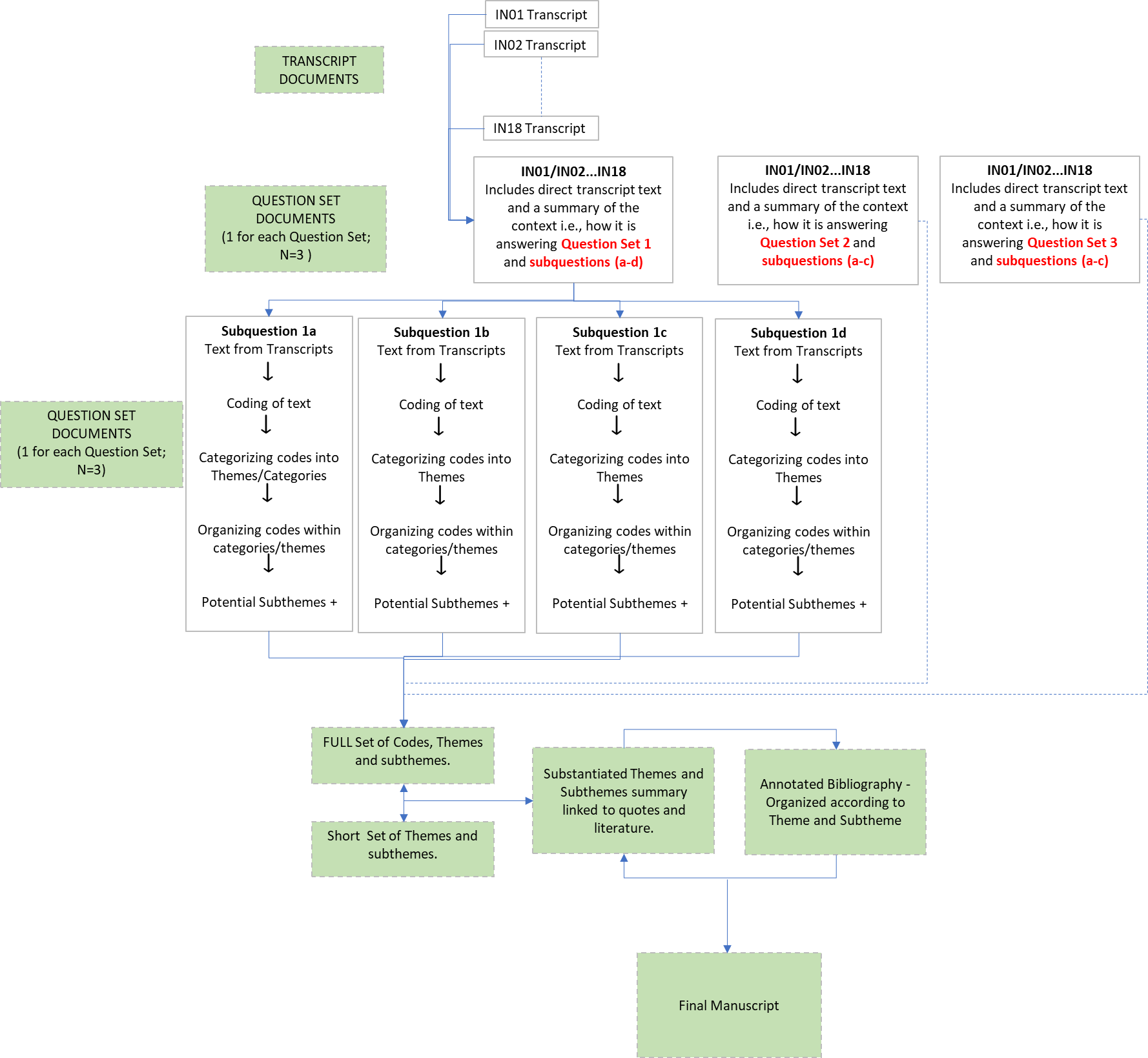


## Supplementary Table 2: Comparison of existing initiatives to a care pathway approach to HTA

|  |  |  | **Alignment with the characteristics of a Care Pathway Approach (Yes, No, Possibly)**  **(Author’s Interpretation)** | | |
| --- | --- | --- | --- | --- | --- |
| **Initiative**  **[Organization]** | **Description** | **Illustrative Quote** | **A) Considers multiple interventions** | **B) Manages diseases vs. manages intervention(s)** | **C) Dynamically considers the relationships between interventions** |
| Lifecycle HTA  (Health Technology Management, Health Technology Reassessment)  **[National and International HTA community]** | *“Life-cycle HTA endeavours to assess the health, economic, and societal impacts of a technology across its life-cycle, from research and development to obsolescence.”(1)*  *“The LC-HTA framework builds upon standard HTA concepts and methods and proposes adaptations that will lead to better outcomes for all stakeholders.”(2)*  *“HTR is contingent on collaboration with diverse stakeholders, such as clinicians, to optimize the value of care and increase appropriateness. This may be achieved by decreasing, increasing, or maintaining current levels of use.”(3)* | *"I think the frame of what you're talking about to me does really fit in the life cycle" (IN16)* | No  Focus is on a specific intervention. | No  Focus is on the intervention not the disease. | Possibly  Could involve reassessing the evidence over time in the context of other interventions. |
| Condition-level reviews  **[CADTH]** | *“A Condition Level Review is an assessment of the evidence on a range of health technologies and emerging issues on all aspects of the condition, including prevention, identification, treatment, and management”(4)* | *"It* [condition level reviews] *was to look at the entire pathway and different interventions along that pathway, but still not quite looking at how they fit together or the impact of one on another." (IN18)* | Yes  However, interventions are assessed in isolation. | Yes | No  Not intended to look at interactions or relationships between interventions. |
| Therapeutic class reviews  **[CADTH]** | *“A Therapeutic Review is an evidence-based review of publicly available sources regarding a therapeutic category of drugs (e.g., antihypertensive agents) or a class of drugs (e.g., angiotensin-converting enzyme inhibitors [ACEIs]) in order to support drug listing and policy decisions, as well as encouraging the optimization of drug therapy.” (5)* | *"But it's still left me thinking this could be something more than just a therapeutic class review…[it] didn't get into prevention, diagnosis, lab tests, rehabilitation." (IN16)* | Yes  However only looking at pharmaceutical interventions. | No  Focus is still intended to be on the management of interventions within a specific therapeutic class. | NA |
| Optimal and appropriate use  **[CADTH]** | *“CADTH and CWC [Choosing Wisely Canada] convened an independent panel of clinicians, patient representatives, and health policy experts to assess and prioritize low-value health care recommendations in the context of post-pandemic health systems. The panel voted and deliberated on these recommendations and highlighted the ones that, based on their assessment, could have the most impact in reducing low-value care in health care systems across Canada after the pandemic.” (6)* | *"the partnership with choosing wisely. I think that's a good start." (IN16)* | No  Not necessarily unless multiple low-value care is identified. | No  Focus is on low-value care regardless of disease. | No  Focus is on low-value care regardless of interactions with other interventions. |
| Mega-analyses  **[Ontario Health]** | *“MAS [Medical Advisory Secretariat] has increasingly been asked to take on broad system-wide, comprehensive reviews of important issues such as aging in the community, management of diabetes, management of COPD and approaches to seizure disorders amenable to surgical treatment.”(7)* | *“We did, many years ago something, you know, looking at, you know, at the pathway of care, looking at all the technologies within. I don't know if we achieved anything through it, you know.” (IN03)* | Yes  However, may not include preventative interventions or pharmaceutical interventions given the mandate of the provincial organization. | Yes  However, topics could also be broader than a specific disease (e.g., aging in the community). | Possibly  However, recommendations may not go as far as exploring the impact of one recommended intervention on another. |
| Health Evidence Reviews  **[Alberta Health]** | “*Health evidence reviews use a research method called health technology assessment. This research is done to help determine what health care technologies, services, and models of care are suitable and sustainable for a given population. Reviews examine how health care services can be delivered better in a certain part of the health care system, or for a specific disease or condition. This is preferred to evaluating individual technologies and services for adoption.”* (8) | *"…broader you know sort of assessments…call them health evidence reviews rather than HTA's now." (IN13)* | Possibly  If the HTA finds that the optimal model is a combination of interventions, these interventions would be considered. | Yes  However, topics could also be broader than a specific disease (e.g., urgent care models). | Possibly  However, the focus of the reviews is on optimal system implementation. |
| OncoSim model  **[Canadian Partnership Against Cancer]** | *“The OncoSim microsimulation model platform, with its suite of specific cancer models, was conceived as a tool to augment conventional information resources for population-level Canadian decision-making, within the reality of thirteen jurisdictional health care systems.”(9)* | *“You can create a model where you start at time 0 and that's what honestly some of the oncoSIM model out of CPAC starts at birth and grows at tumor inside the woman and some of those, you know, some of these unchecked growth cells go on to a diagnosis of cancer and some don't. And so they sort of model what will happen.” (IN08)* | Yes | Yes  However, the focus of onco-sim is on the decision-modeling component of HTA. | Yes  Can consider combinations of interventions. |
| Whole disease modeling  **[University of Sheffield, UK; Tappenden et al.]** | “Whole Disease Modeling—the notion of modeling the “bigger picture” by simulating whole disease and treatment pathways within a single model. The distinguishing characteristics of Whole Disease Modeling that set it apart from the conventional piecewise approach are the wider disease-level model scope of the model and its structural ability to evaluate alternative interventions across the disease pathway within a single mathematical framework.”(10) | *"He had attempted to do exactly the type of thing you're talking about and did it for oral cancer. He kind of followed on from Paul Tappenden in the UK" (IN12)* | Yes | Yes  However, the focus of whole disease modeling is on decision- modeling component of HTA. | Yes |
| Diagonal approach  **[IHE, Alberta, Canada; Kirwin et al.]** | *“Vertical interventions typically target well-defined patient groups and/or disease outcomes. Examples include a drug used to treat a specific patient group, or a surgical procedure… Horizontal interventions are those that are aimed at improving the overall structure and function of the health system. Horizontal interventions act on platforms: facilities, workforces, policies, and other physical or abstract structures impacting the delivery of vertical interventions...The diagonal approach can be interpreted as the identification of the optimal combination of horizontal and vertical interventions to implement.”(11)* | *“…all of the costs of running the genomic testing program are assumed to be related to a particular cancer type and directed therapies for them rather than well, actually there's an infrastructure cost that applies across all. " (IN13)* | Yes  Interventions could also include horizontal interventions – i.e., those that aim to improve the structure and function of the health system. | Possibly  However, focus is on cost-effectiveness analyses, and is not necessarily disease specific. | Yes  Focuses on optimizing between interventions. |
| Interactive technologies  **[University of Oxford; Dakin and Gray]** | “Interactions (i.e.,  non-additive effects, whereby the effect of giving 2 interventions simultaneously does not equal the sum of their individual effects) are the defining feature of mutually exclusive alternatives: treatments cannot be considered independent  if the costs and/or benefits of one treatment are affected by the other treatment.”(12) | *"she's been exploring and coming up or trying to come up with a template for determining what interactions are important and what are not" (IN09)* | Yes | Possibly  However, focus is on cost-effectiveness analyses, and is not necessarily disease specific. | Yes |
| Considering opportunity cost  **[McMaster University, Canada; Birch and Gafni]** | “…the economic methods used to appraise  interventions must incorporate explicit recognition of the opportunity costs of  new interventions.”(13) | *“…his big criticism was the lack of consideration of opportunity cost. You know, like do you making one off decisions you're not actually like placing it in the context of like what's being foregone" (IN14)* | Yes | Possibly  However, focus is on cost-effectiveness analyses, and is not necessarily disease specific. | Yes |
| Program Budgeting and Marginal Analysis  **[UBC, Canada; Mitton et al.]** | *“PBMA is an approach for setting priorities which has been reported to be used in health organizations mainly in Britain, Australia, New Zealand and Canada. The approach is based on the same principles as economic evaluation, but has been argued to be more pragmatic and is applicable at various levels within health organizations…key is in ensuring that opportunity cost and the margin are brought to the fore of the decision making process.”(14)* | *"let's pick out a series of interventions that we think are worth considering for one reason or another.….say, prevention interventions and then you can you put them in the same kind of example with some treatment based interventions and look at that issue should you be swapping between portfolios" (IN12)* | Yes  Priority setting from a list of interventions. | Possibly  Depends on the scope of the portfolio of interventions. | Possibly  Focus is on prioritizing interventions within a given budget, not necessarily the relationship between the interventions. |
| Assessment of Cost-Effectiveness Models  **[Deakin University, Australia; Carter et al.]** | “The decision context for most ACE evaluations has been the possible adoption Australia-wide of options to improve the efficiency of current health services and to inform policy makers about the best bundle of interventions, given alternative levels of budget availability.”(15) | *"he has something called the assessment of cost effectiveness models and he uses like the global burden of disease DALY type approach to look at more macro style decision making" (IN12)* | Yes  Priority setting from a list of interventions. | Possibly  Depends on the scope of the portfolio of interventions. | Possibly  Focus is on cost and cost-effectiveness to inform decision-making. Could be used within a PBMA framework. |

# References

1. Regier DA, Pollard S, McPhail M, Bubela T, Hanna TP, Ho C, et al. A perspective on life-cycle health technology assessment and real-world evidence for precision oncology in Canada. npj Precision Oncology. 2022;6(1):76.

2. Kirwin E, Round J, Bond K, McCabe C. A Conceptual Framework for Life-Cycle Health Technology Assessment. Value in Health. 2022;25(7):1116-23.

3. Soril LJ, MacKean G, Noseworthy TW, Leggett LE, Clement FM. Achieving optimal technology use: A proposed model for health technology reassessment. SAGE Open Med. 2017;5:2050312117704861.

4. CADTH. Post‒COVID-19 Condition: A Condition-Level Review 2022 [Available from: <https://www.cadth.ca/post-covid-19-condition-condition-level-review>.

5. CADTH. Therapeutic Review Framework 2020 [Available from: <https://www.cadth.ca/therapeutic-review-framework#:~:text=A%20Therapeutic%20Review%20is%20an,as%20well%20as%20encouraging%20the>.

6. Basharat S, Born K. Using Health Care Resources Wisely After the COVID-19 Pandemic: Recommendations to Reduce Low-Value Care. Canadian Journal of Health Technologies. 2021;1(5).

7. Health Quality Ontario. Process review and evaluation of the evidence based health technology analysis program in  Ontario, including the Medical Advisory Secretariat (MAS)  and the Ontario Health Technology Advisory Committee (OHTAC). 2012.

8. Alberta Health. Health Evidence Reviews 2022 [Available from: <https://www.alberta.ca/health-evidence-reviews.aspx#jumplinks-2>.

9. Gauvreau CL, Fitzgerald NR, Memon S, Flanagan WM, Nadeau C, Asakawa K, et al. The OncoSim model: development and use for better decision-making in Canadian cancer control. Curr Oncol. 2017;24(6):401-6.

10. Tappenden P, Chilcott J, Brennan A, Squires H, Stevenson M. Whole disease modeling to inform resource allocation decisions in cancer: a methodological framework. Value Health. 2012;15(8):1127-36.

11. Kirwin E, Meacock R, Round J, Sutton M. The diagonal approach: A theoretic framework for the economic evaluation of vertical and horizontal interventions in healthcare. Social Science & Medicine. 2022;301:114900.

12. Dakin H, Gray A. Decision Making for Healthcare Resource Allocation: Joint v. Separate Decisions on Interacting Interventions. Med Decis Making. 2018;38(4):476-86.

13. Birch S, Gafni A. Economists' dream or nightmare? Maximizing health gains from available resources using the NICE guidelines. Health Econ Policy Law. 2007;2(Pt 2):193-202.

14. Mitton C, Donaldson C. Health care priority setting: principles, practice and challenges. Cost Effectiveness and Resource Allocation. 2004;2(1):3.

15. Carter R, Vos T, Moodie M, Haby M, Magnus A, Mihalopoulos C. Priority setting in health: origins, description and application of the Australian Assessing Cost-Effectiveness initiative. Expert review of pharmacoeconomics & outcomes research. 2008;8(6):593-617.
